# Reduced Efficacy of Selection on a Young Z Chromosome Region of *Schistosoma japonicum*

**DOI:** 10.1093/gbe/evaf021

**Published:** 2025-02-06

**Authors:** Andrea Mrnjavac, Beatriz Vicoso

**Affiliations:** Institute of Science and Technology Austria, Klosterneuburg 3400, Austria; Institute of Science and Technology Austria, Klosterneuburg 3400, Austria

**Keywords:** sex chromosomes, population genomics, molecular evolution

## Abstract

Sex-linked and autosomal loci experience different selective pressures and evolutionary dynamics. X (or Z) chromosomes are often hemizygous in males (or females), as Y (or W) chromosomes often degenerate. Such hemizygous regions can be under greater efficacy of selection, as recessive mutations are immediately exposed to selection in the heterogametic sex leading to faster adaptation and faster divergence on the X chromosome (the so-called Faster-X or Faster-Z effect). However, in young nonrecombining regions, Y/W chromosomes often have many functional genes, and many X/Z-linked loci are therefore diploid. The sheltering of recessive mutations on the X/Z by the Y/W homolog is expected to drive slower adaptation for diploid X/Z loci, i.e. a reduction in the efficacy of selection. While the Faster-X effect has been studied extensively, much less is known empirically about the evolutionary dynamics of diploid X or Z chromosomes. Here, we took advantage of published population genomic data in the female-heterogametic human parasite *Schistosoma japonicum* to characterize the gene content and diversity levels of the diploid and hemizygous regions of the Z chromosome. We used different metrics of selective pressures acting on genes to test for differences in the efficacy of selection in hemizygous and diploid Z regions, relative to autosomes. We found consistent patterns suggesting reduced Ne, and reduced efficacy of purifying selection, on both hemizygous and diploid Z regions. Moreover, relaxed selection was particularly pronounced for female-biased genes on the diploid Z, as predicted by recent theoretical work.

**Significance**

Faster adaptive evolution is a well-known feature of several differentiated X (or Z) chromosomes. On the other hand, recent theoretical work suggests that slower adaptation and relaxation from selective constraints are expected in young X/Z-linked regions. The *Schistosoma japonicum* Z chromosome contains both a well-differentiated region whose homologous region on the W has degenerated, and a very young nondifferentiated, but nonrecombining region with a nondegenerated homologous region on the W. This provides an ideal opportunity to compare evolutionary dynamics in the young Z region, the differentiated Z region, and the autosomes in the same species. Our results provide direct evidence of reduced efficiency of selection in the young Z region, likely due to sheltering by the functional gene copy on the W, in line with theoretical predictions. Together with other recent empirical findings, our results suggest that relaxation of selective constraints could be a common feature of young X/Z chromosomes, which has important implications for sex chromosome evolution.

## Introduction

Sex chromosomes, such as the X and Y of mammals, or the Z and W of birds, originate from standard pairs of autosomes. After they are coopted for sex determination, the two chromosomes typically stop recombining and start diverging from each other (Furman et al. 2020). This leads them to evolve differently from autosomes. The most striking aspect of this is the progressive degeneration of the nonrecombining Y/W that is observed in many clades (Charlesworth 2021). However, it has become increasingly appreciated that evolutionary rates on the X chromosome (or Z, but explained in terms of the X for simplicity) are also shaped by unusual evolutionary pressures. All else being equal, the effective population size of the X chromosome is three-fourths the autosomal effective population size, while Y chromosomes have a population size of only one-fourth the autosomal one (Vicoso and Charlesworth 2009). Both X and Y chromosomes exhibit sex-biased transmission: the X resides in females two-thirds of the time, while the Y is in males 100% of the time (Furman et al. 2020). Furthermore, the degeneration of the Y chromosome (Bachtrog 2013) leaves X-linked loci hemizygous in males. Selection is more efficient for mutations affecting male fitness on X-linked loci with degenerated Y gametolog (“hemizygous” X-linked loci) despite its reduced effective population size, as recessive mutations are always exposed to selection in males. On the other hand, on the autosomal loci recessive mutations are mostly found in heterozygous form and their effect is masked by the dominant allele. This should lead to higher rates of adaptive evolution on the X chromosome than autosomes if new beneficial mutations are on average recessive, and consequently faster divergence, a hypothesis known as the Faster-X effect (Charlesworth et al. 1987; Vicoso and Charlesworth 2006). Support for the Faster-X effect comes from the observation of elevated dN/dS on the X chromosome, or elevated values of *α*, the inferred proportion of nonsynonymous divergent sites that were fixed by positive selection (Meisel and Connallon 2013), in various clades.

There is empirical evidence for high rates of nonsynonymous evolution on the X in mammals and *Drosophila*, and on the Z in birds and arthropods (Mank, Nam et al. 2010; Meisel and Connallon 2013; Charlesworth et al. 2018; Mongue et al. 2022). However, evidence suggests that this is not always driven by increased rates of adaptation. While there is evidence of increased rates of adaptive divergence on various X chromosomes (Ávila et al. 2014; Campos et al. 2014; Garrigan et al. 2014; Kousathanas et al. 2014; Veeramah et al. 2014; Charlesworth et al. 2018), the Faster-Z effect has been interpreted as being the result of stronger drift on the Z chromosome of several species (Mank, Nam et al. 2010; Hayes et al. 2020; Chase et al. 2024; Mongue and Baird 2024, but see Wanders et al. 2024). This is possibly due to the fact that the Z spends more time in males: males usually have a higher variance in reproductive success, resulting in the more extreme reduction in the effective population size for the Z chromosome than for the X (Vicoso and Charlesworth 2009; Mank, Vicoso et al. 2010). In smaller populations, a higher proportion of mutations entering the population is effectively neutral, contributing to faster nonadaptive evolution (Ohta 1992; Mank, Nam et al. 2010; Mank, Vicoso et al. 2010). On the other hand, evidence of faster and more adaptive evolution on the Z was found in some Lepidoptera, which typically have a larger population size than birds (which may make the Z chromosome less sensitive to the reduction in the effective population size) (Mongue et al. 2022; Villavicencio et al. 2024).

X-linked loci in young nonrecombining regions, which still have a nondegenerated homologous region on the Y chromosome, are not hemizygous, but diploid in males, as they have a functional, albeit nonrecombining, gametolog on the Y. Unlike loci on older, male-hemizygous X chromosomes, such “male diploid X” loci are not expected to adapt faster than autosomal loci. For simplicity, we refer to X/Z-linked genes based on their zygosity state in the heterogametic sex, i.e. “diploid” if they have a homolog on the Y/W, and “hemizygous” if they do not. New mutations that arise on a diploid X region are always heterozygous in males, and, if (partly) recessive, are (partially) sheltered from selection by the functional copy on the Y. This is expected to cause reduced efficiency of selection in males on the diploid X region, slower adaptation of male-important genes, and accumulation of deleterious mutations on male-important genes (Mrnjavac et al. 2023).

The evolutionary patterns of young nonrecombining regions on the X or Z have been studied less often than well-differentiated sex-linked regions, as population data is needed to detect very young nonrecombining regions with nondegenerated Y counterparts (Vicoso 2019; Darolti et al. 2022), but a few have found some support for reduced efficiency of selection in the early stages of X/Z differentiation. Neo-X regions (with the corresponding Y chromosomes showing intermediate levels of degeneration) in several *Drosophila* species experience accelerated pseudogenization, driven by the loss of male-important genes (Nozawa et al. 2016; Nozawa et al. 2021). In the plant *Silene latifolia* there is evidence of relaxed purifying selection on young X-linked genes with a nondegenerated Y homolog (Krasovec et al. 2018). Recently, a study in the butterfly genus *Leptidea* provided direct empirical evidence of reduced efficiency of selection for female-biased and unbiased genes on the young nonrecombining region of the Z chromosome with a nondegenerated W (Höök et al. 2024). Some studies, on the other hand, have found similar rates of divergence for diploid X/Z genes as for (pseudo)autosomal genes. The young X-linked region of the plant *Salix dunni* is enriched for transposable elements and pseudogenes, but divergence of X-linked genes is similar to the autosomal divergence, possibly because the X-linked region is very young and there was no time for nonadaptive substitutions to accumulate (He et al. 2021). Similarly, in *Sylvioidea* songbirds, there is no difference in evolutionary rates between the neo-Z and autosomes (Leroy et al. 2021). Darolti et al. (2023) further showed that while Faster-X correlates with hemizygosity in various species of poeciliid fishes, no evidence of increased drift or differences in divergence rates could be detected between diploid X chromosomes and their respective autosomes. Therefore, the broad relevance of the Slower-X effect in taxa with young sex-linked regions is still to be fully explored.

Blood flukes (genus *Schistosoma*) are a promising model for studying the evolutionary dynamics of sex-linked regions of different ages. While they all share an ancestral pair of ZW chromosomes, the nonrecombining part of the sex chromosomes has been expanded independently in different lineages (Picard et al. 2018). A very young nonrecombining region of the Z chromosome has been recently identified in the Asian species *S. japonicum* (Elkrewi et al. 2021; Xu et al. 2023). This region has over 700 genes (ZW dS < 0.085). A copy number variation analysis did not detect an excess of female gene loss in this region compared with the autosomes, suggesting that no full degeneration of W-linked genes has occurred (Elkrewi et al. 2021). Furthermore, W genes in this region only show a modest increase in their ratio of nonsynonymous to synonymous divergence compared with their Z counterparts (Elkrewi et al. 2021). Finally, W-linked gene expression levels are slightly lower than that of their Z-homologues, but not significantly so, again suggesting functionality of identified W genes (Elkrewi et al. 2021). It should be noted that these analyses only considered the most diverged Z:W homolog pairs in this young sex-linked region; for most gene pairs, no difference beyond an excess of female:male SNP differentiation could be detected, again supporting the idea that overall this part of the W has stopped recombining but has yet to lose functionality. We therefore expect the homologous region on the Z chromosome to be under reduced efficiency of selection in females, compared with autosomes and hemizygous Z (Mrnjavac et al. 2023). Here, we use publicly available comparative (Protasio et al. 2012; Luo et al. 2022), population (Luo et al. 2022) and expression data (Wang et al. 2017) to test these predictions.

## Results

### Hemizygous and Diploid Regions of the Z Chromosome

**Fig. 1.**
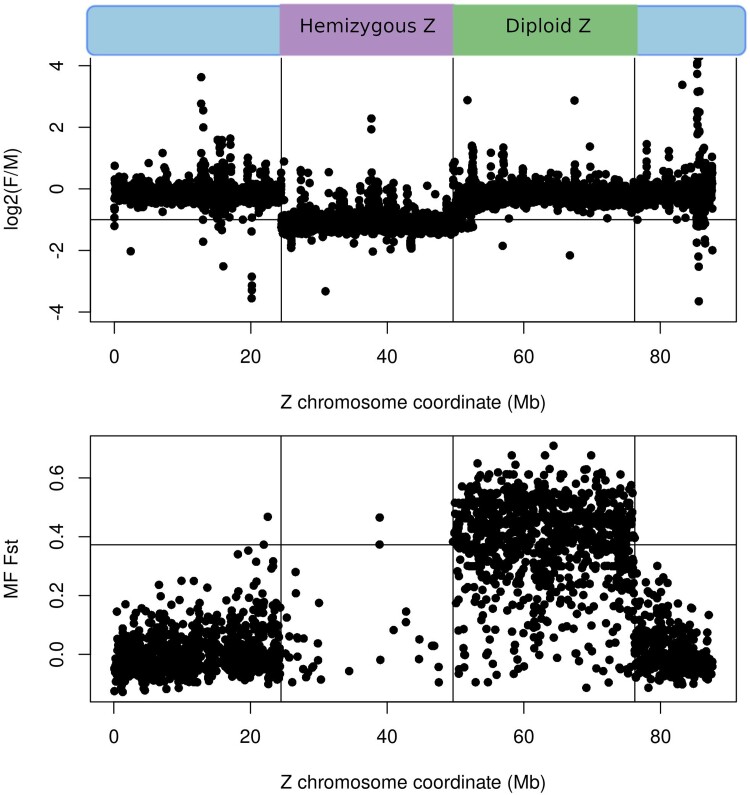
*Schistosoma japonicum* Z chromosome strata: hemizygous and diploid Z regions. Upper panel: Log2(Female coverage/Male coverage) along the Z chromosome. Hemizygous Z region has male coverage that is two times the female coverage. Lower panel: Male-to-Female *F*_st_ along the Z chromosome. Diploid Z region has equal coverage in males and females, but has high levels of Male-to-Female *F*_st_. Two terminal PAR have equal coverage in males and females and low *F*_st_ between male and female samples.

Elkrewi et al. (2021) and Xu et al. (2023) recently described evolutionary strata of different ages along the Z chromosome of *S. japonicum*, and in particular the presence of a large section of the ZW pair that no longer recombines, but still exists on the W. However, a highly fragmented genome was used in Elkrewi et al. (2021), and no population genomics data was used to infer young nonrecombining regions in Xu et al. (2023). We therefore set out to define precise boundaries of the Z chromosome regions with nondegenerated W counterpart (diploid Z) and degenerated W counterpart (hemizygous Z) on the published chromosome-level assembly of *S. japonicum* (Luo et al. 2022). Using both coverage patterns and genetic differentiation between a population of males and females, we recovered large contiguous hemizygous and nonrecombining but diploid Z regions (Elkrewi et al. 2021; Xu et al. 2023) (Fig. 1). A large region where female coverage is consistently half of male coverage suggests degeneration of the homologous region of the W chromosome, i.e. this Z chromosome region is hemizygous in females. A second region shows no difference in coverage between males and females, but shows a high level of genetic differentiation between the Z and W, measured as male-to-female *F*_st_, consistent with a recent loss of recombination between the Z and W, and a nondegenerated homologous region on the W chromosome. The hemizygous and diploid Z regions contain, respectively, 703 and 624 genes. There are also two terminal pseudoautosomal regions (PARs) with 519 and 270 genes. PARs exhibit equal coverage in males and females and no differentiation between male and female reads, which suggests the presence of recombination between Z and W in these regions.

### Lower Effective Population Size on the Z Chromosome

Since males have two Z chromosomes but females only have one (compared with two sets of autosomes in each sex), the expected effective population size of the Z is three-fourths of that of the autosomes. We estimated total genetic pairwise diversity (*π*) from a population of 48 males from six sampling locations (the original dataset contained the data from eight sampling locations, but two were excluded from the analysis, see below), and used it to infer the effective population size of the hemizygous and diploid Z regions relative to that of the autosomes in *S. japonicum*. Both hemizygous and diploid Z regions show lower than expected nucleotide diversity compared with autosomes, with a Z/A ratio of median nucleotide diversity of 0.37 for the diploid Z region and 0.09 for the hemizygous Z region (*P* < 1e^−10^, *P* < 1e^−10^, respectively, supplementary fig. S1, Supplementary Material online), which suggests that the effective population size of the Z chromosome could be even lower than three-fourths of the autosomal effective population size. Similar estimates were obtained when only synonymous sites were used to calculate diversity (Z:A ratios of 0.395 and 0.235 for the diploid and hemizygous regions). Given the apparent young age of the diploid Z region, we took advantage of the reduced *π* on the diploid Z to check that the loss of ZW recombination was found in every population. Two island populations (Taiwan and the Philippines) had extremely reduced levels of overall diversity and were excluded from further analysis. In each of the other six populations, the diploid Z region had reduced levels of diversity compared with the autosomes (*P* < 1e^−10^, Mann–Whitney–Wilcoxon test), confirming that it is nonrecombining throughout the geographical range of the species (supplementary fig. S1b, Supplementary Material online). Such a reduction was not observed in the pseudoautosomal region (*P* > 0.1).

### Reduced Efficacy of Purifying Selection on Both the Hemizygous and Diploid Z Regions

**Fig. 2.**
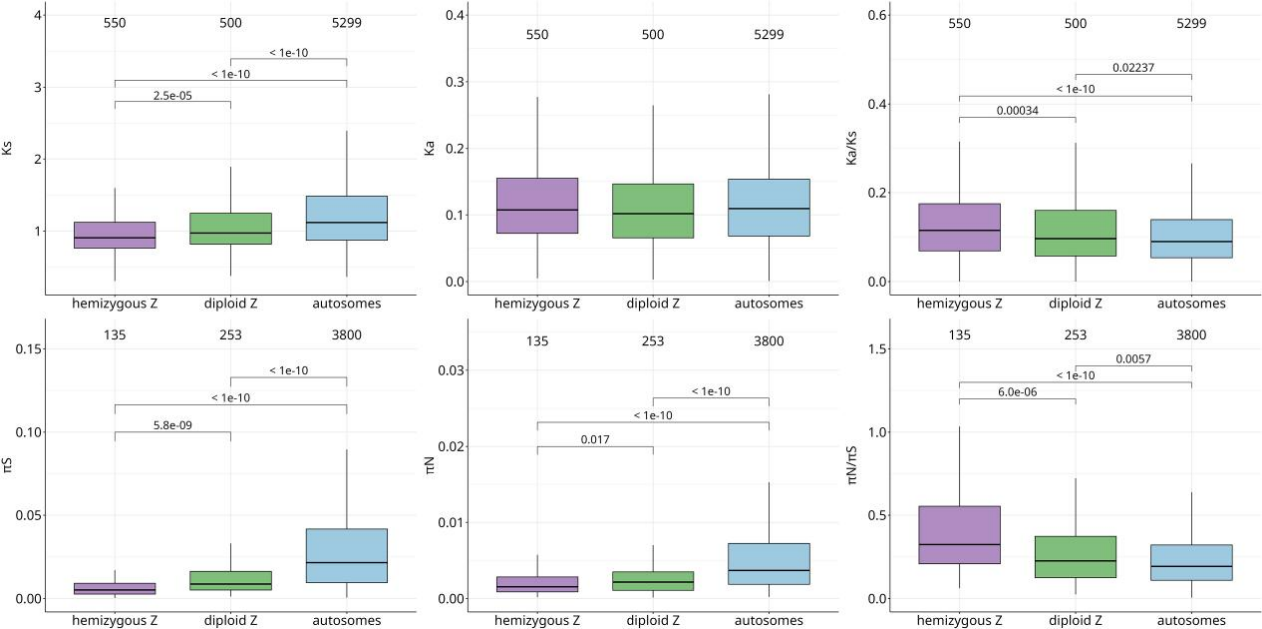
Synonymous (*K*_s_) and nonsynonymous rates of divergence (*K*_a_) between *S. japonicum* and *S. mansoni*, and their ratio (*K*_a_/*K*_s_), synonymous (*π*_S_) and nonsynonymous diversity (*π*_N_) in a population of males, and their ratio (*π*_N_/*π*_S_) for hemizygous and diploid Z and autosomes. Numbers of genes are labeled above boxplots.

We first measured the divergence between *S. japonicum* and the closely related species *Schistosoma mansoni* to estimate synonymous (*K*_s_) and nonsynonymous (*K*_a_) substitution rates per gene, i.e. the number of synonymous substitutions per synonymous site and the nonsynonymous substitutions per nonsynonymous site per gene. Figure 2 shows the distribution of *K*_a_/*K*_s_ per gene for the hemizygous Z, the diploid Z and the autosomes. Distributions of *K*_a_/*K*_s_ per gene in different genomic regions were statistically compared using the Wilcoxon–Mann–Whitney test. *K*_a_/*K*_s_ is significantly higher on the hemizygous Z (hZ) compared with both autosomes (A) and to the diploid Z (dZ) [median (hZ) = 0.1152, median (dZ) = 0.0966, median (A) = 0.0901, *P* = 1.327e^−13^, *P* = 0.0003441, respectively], while diploid Z genes show a slight increase compared with the autosomes (*P* = 0.02237). *K*_a_/*K*_s_ on PARs was somewhat lower than on the autosomes [median (PAR1) = 0.0801, median (PAR2) = 0.0876, although the difference was significant only for PAR1, *P* = 0.0007, supplementary fig. S3, Supplementary Material online]. Synonymous divergence is significantly lower on the Z chromosome, with hemizygous Z exhibiting the lowest synonymous divergence [median (hZ) = 0.9071, median (dZ) = 0.9729, median (A) = 1.121, hZ versus A: *P* < 2.2e^−16^, dZ versus A: *P* = 1.656e^−11^, hZ versus dZ: *P* = 2.493e^−5^]. Overall these results support the faster protein divergence of Z-linked genes compared with the autosomes.

In order to investigate whether this fast evolution of Z-linked genes was driven by an increase in positive selection or by a decrease in the efficacy of purifying selection, we obtained estimates of synonymous and nonsynonymous polymorphism across the sampled populations (excluding the two that did not harbor any diversity). Diversity levels in different genomic regions were compared with the Wilcoxon–Mann–Whitney test. Both haploid and diploid Z regions have higher levels of nonsynonymous to synonymous diversity compared with the autosomes [median (hZ) = 0.3242, median (dZ) = 0.2295, median (A) = 0.1942, hZ versus A: *P* = 5.9e^−15^, dZ versus A: *P* = 0.0057, Fig. 2]. This is in line with the reduced effective population size and the resulting reduced efficiency of selection in removing slightly deleterious mutations from the population. On the other hand, PARs exhibit somewhat lower *π*_N_/*π*_S_ compared with autosomes, though the difference is significant only for PAR2 (*P* = 0.00016) [median (PAR1) = 0.1816, median (PAR2) = 0.1485, supplementary fig. S3, Supplementary Material online]. We also calculated *α* per gene, a commonly used measure of adaptive evolution based on the McDonald-Kreitman test (McDonald and Kreitman 1991; Smith and Eyre-Walker 2002; Charlesworth and Charlesworth 2010). Positive *α* values suggest positive selection, while negative *α* values mean there is an excess of nonsynonymous polymorphisms segregating in the population. This excess is usually caused by segregating slightly deleterious mutations, that is, lower efficiency of selection, or, balancing selection (Charlesworth and Charlesworth 2010, Chapter 6.4). We removed rare polymorphic sites (with minor allele frequency below 15%) from the analysis to minimize the contribution of deleterious mutations segregating at low frequencies (Fay et al. 2001). We also removed the genes with no polymorphism for the downstream analysis, which greatly reduced the number of genes: in the hemizygous Z region up to 80% of the genes did not exhibit any polymorphism after filtering out rare variants, while in the diploid Z region and autosomes, from 20% to 60% of genes exhibited no polymorphisms. This reflects extremely low levels of nonsynonymous and synonymous polymorphisms segregating on the hemizygous Z region (Fig. 2), congruent with the extreme reduction in the population size for hemizygous Z compared with the rest of the genome (supplementary fig. S1, Supplementary Material online). The small number of genes in some categories, especially on hemizygous Z, greatly reduced our statistical power. It should also be noted that our values of *α* likely underestimate the true proportion of nonsynonymous substitutions fixed by positive selection. However, we are interested in relative differences in the strength of selection in different genomic regions. A recent study (Al-Saffar and Hahn 2022) showed that the Fay et al. (2001) approach of filtering out low frequency variants underestimates the true value of *α*, but accurately reflects differences between the X chromosome and autosomes. Furthermore, *α* values for the diploid Z region should be interpreted with caution, as this region only recently became diploid, such that its divergence reflects both Z-linked and autosomal evolutionary patterns. In agreement with purifying selection being relaxed on both the hemizygous and diploid Z, genes in both regions showed reduced *α* values compared with autosomal genes [median (hZ) = −0.4922, median (dZ) = −0.0974, median (A) = 0.0721, *P* = 4.5e^−10^ and *P* = 0.005, respectively, supplementary fig. S3, Supplementary Material online]. Once again, the effect was stronger for the hemizygous Z region than for the diploid Z.

In addition to *α*, we calculated a second metric of selection strength, the Direction of Selection (DoS) (Stoletzki and Eyre-Walker 2011), and the results were qualitatively similar (supplementary fig. S5a, Supplementary Material online). The lower values of DoS observed for both hemizygous and diploid Z genes compared with autosomal genes [median (hZ) = −0.0913, median (dZ) = −0.0232, median (A) = 0.0166, *P* = 4.7e^−10^ and *P* = 0.0043] suggest that there is an excess of nonsynonymous polymorphisms that reach high frequencies in the population (as we removed rare variants) on both regions of the Z.

### Different Evolutionary Dynamics of Hemizygous and Diploid Z-linked Sex-biased Genes

Genes with sex-specific functions are expected to evolve differently on hemizygous and diploid Z-linked regions. To test this hypothesis, we used sex-specific patterns of expression as a proxy for function. We measured sex bias in the expression of the *S. japonicum* adult whole body as log2(M/F) expression, where log2(M/F) ≥ 1 corresponds to male-bias and log2(M/F)≤−1 corresponds to female-bias. Supplementary fig. S2, Supplementary Material online shows distributions of sex-biased expression on autosomes, in the diploid Z region and in the hemizygous Z region. The hemizygous Z region is significantly masculinized (*P* < 1e^−10^), in agreement with its incomplete mechanism of dosage compensation (Picard et al. 2018), while the diploid Z region exhibits autosomal patterns of sex-bias distribution (*P* > 0.05), possibly because we are capturing expression from both the Z and W.

**Fig. 3.**
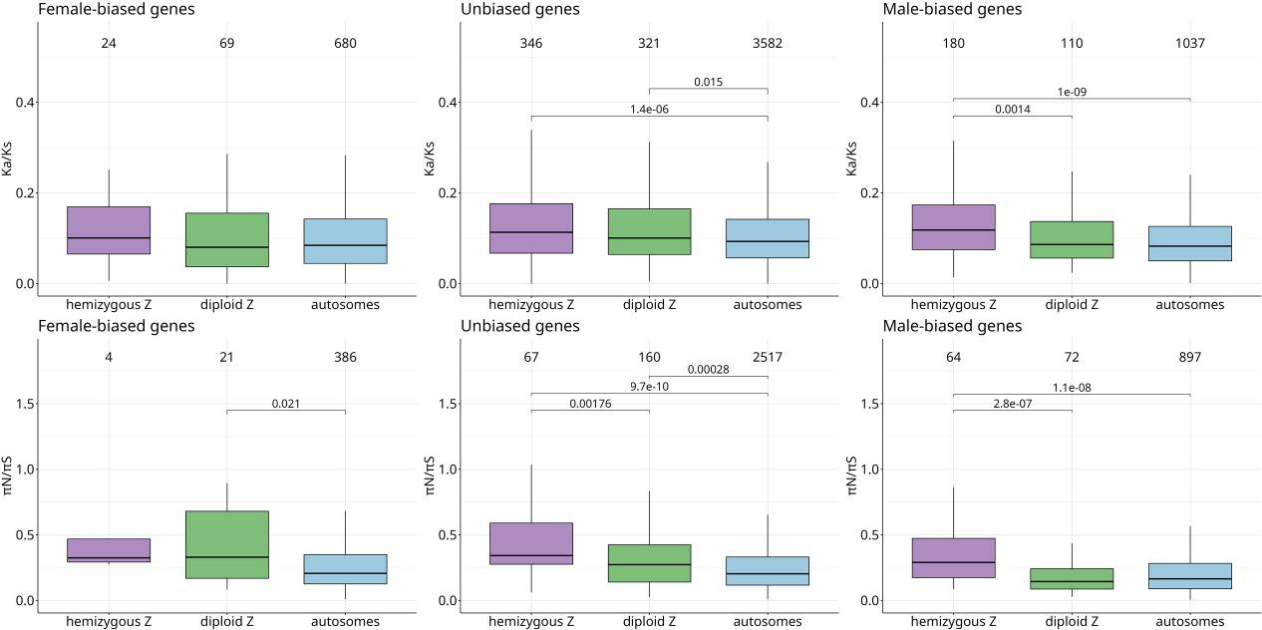
*K*_a_/*K*_s_ and *π*_N_/*π*_S_ for hemizygous and diploid Z and autosomes as a function of sex-bias. Numbers of genes are labeled above boxplots.

**Fig. 4.**
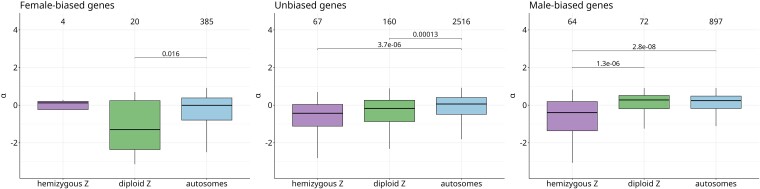
Α as a function of sex-bias and a genomic location. Numbers of genes are labeled above boxplots.

Figure 3 shows nonsynonymous to synonymous substitution rates and genetic diversity as a function of sex-bias and genomic region: hemizygous Z, diploid Z and autosomes. Differences in divergence and diversity patterns between different genomic regions were statistically compared within each category of sex-bias using the Wilcoxon–Mann–Whitney test. Unbiased genes generally follow the trends described above for all genes: both hemizygous Z and diploid Z genes have increased *K*_a_/*K*_s_ [median (hZ) = 0.1137, median (dZ) = 0.1005, median (A) = 0.0932, hZ versus A: *P* = 1.372e-06, dZ versus A: *P* = 0.0151] and increased *π*_N_/*π*_S_ compared with autosomal genes [median (hZ) = 0.3425, median (dZ) = 0.2733, median (A) = 0.2042, hZ versus A: *P* = 9.722e^−10^, dZ versus A: *P* = 0.00028], consistent with reduced efficacy of selection on both parts of the Z. This is also supported by their reduced *α* values compared with autosomal values [Fig. 4, median (hZ) = −0.5102, median (dZ) = −0.2836, median (A) = 0.0217, hZ versus A: *P* = 3.727e^−06^, dZ versus A, *P* = 0.00013].

In the hemizygous Z region, a key prediction is that genes that function primarily in females are expected to be under stronger efficacy of selection than equivalent autosomal genes, potentially leading to higher rates of adaptive divergence. While the small number of female-biased genes on the hemizygous Z (4 genes) makes it impossible to draw strong conclusions, their *α* trended toward higher values than those of autosomal female-biased genes [though not significantly so, median (hZ) = 0.1204, median (A) = −0.0445]. No significant differences were detected for *K*_a_/*K*_s_ or *π*_N_/π_S_, although this may again be due to limited power. Male-biased genes on the hemizygous Z showed higher *K*_a_/*K*_s_ than male-biased genes on autosomes [median (hZ) = 0.1182, median (A) = 0.0827, *P* = 9.992e^−10^], as well as elevated levels of *π*_N_/π_S_ [hZ versus A mb: W = 35139, *P*-value = 3.512 × 10^−14^] and reduced values of *α* [median (hZ) = −0.4840, median (A) = 0.2307, *P* = 2.787e^−08^], consistent with a primary role of relaxed purifying selection on the hemizygous Z.

On the diploid Z, the expectation is that female-biased genes should be under strongly reduced efficacy of selection. Neither female-biased nor male-biased genes on the diploid Z showed a significant difference in *K*_a_/*K*_s_ when compared with their respective autosomal controls [median (dZ, male-biased) = 0.0863, median (dZ, female-biased) = 0.0802]. While *π*_N_/*π*_S_ did not differ between diploid Z and autosomal male-biased genes [median (dZ) = 0.1445, median (A) = 0.1653], suggesting the two are under similar selective pressures, female-biased genes on the diploid Z had higher *π*_N_/*π*_S_ [median (dZ) = 0.3302, median (A) = 0.2077, *P* = 0.0207] and lower α than their autosomal counterparts [median (dZ) = −1.374012, median (A) = −0.0445, *P* = 0.0164] (Figures 3 and 4). This is generally in line with our predictions that mutations can freely accumulate on female-biased genes in diploid Z, as they are sheltered from selection by the functional gametolog on the W (Mrnjavac et al. 2023).

## Discussion

The sex chromosomes of *S. japonicum*, with their clearly distinguishable female-hemizygous Z and diploid Z regions, that is, regions with degenerated W and with nondegenerated W homologs (Elkrewi et al. 2021; Xu et al. 2023), provide an ideal opportunity to study the evolutionary dynamics of young and old sex-linked regions in the same species. Our results suggest that the effective population size (Ne) of the Z chromosome in *S. japonicum* is much lower than the expected three-fourths of the autosomal effective population size. Since neutral diversity levels are a function of N_e_ times the mutation rate *μ*, it is possible that the difference is driven by differences in *μ*. To account for this, we also compared the distribution of *π*/*K*_s_, which should control for the mutation rate. Values for the Z were still lower than three-fourths of those of the autosomes (hZ/A = 0.2584, dZ/A = 0.4290, supplementary fig. S6, Supplementary Material online), suggesting a true reduction in Ne. The effective population size of Z chromosomes is expected to typically be smaller than the effective population size of X chromosomes, because Z chromosomes spend most of the time in males, and males often have a larger variance of reproductive success, which decreases their effective population size (Caballero 1995; Charlesworth 2001; Laporte and Charlesworth 2002; Vicoso and Charlesworth 2009; Mank, Vicoso et al. 2010). While the variance in reproductive success of males and females of *S. japonicum* is not known, populations of adults are typically male-biased (Beltran and Boissier 2010). Given the largely monogamous reproductive mode of schistosome parasites (Beltran and Boissier 2008), this may lead to a substantial proportion of males remaining unpaired, thereby increasing the variance in their reproductive success.

Interestingly, the N_e_ of the hemizygous Z is lower than the N_e_ of the diploid Z. One possibility to explain this is that the hemizygous Z region has been nonrecombining for a longer amount of time: if loss of recombination with the W occurred very recently, the diploid Z may still not have lost all the standing variation that it harbored when it was a pseudoautosomal region. This is however unlikely to fully explain the pattern, as the reduction in N_e_ following a decrease in population size should occur fairly rapidly (as the long-term N_e_ is simply the harmonic mean of the population sizes over generations, Nei and Tajima 1981; *K*_a_linowski and Waples 2002). The lower N_e_ observed on the Z chromosome could also be due to the stronger effect of linked selection. The effect of linked selection should be particularly strong on the hemizygous Z, due to recessive mutations being exposed to selection. While we did not detect evidence of stronger positive selection on the hemizygous Z than on the autosomes, a recent study did detect a few loci under strong selection (Zhou et al. 2024), which may have contributed to reducing its genetic diversity. Additionally, it is possible that the hemizygous Z region does not recombine even in males (or has very low recombination rates), which would further reduce diversity. While no linkage map is available for *S. japonicum*, the Z-specific region of its close relative *S. mansoni*, which is partly shared with *S. japonicum*, has normal levels of recombination in males (Criscione et al. 2009). It therefore seems likely that a combination of factors drives the strong reduction in N_e_ that we observe.

Consistent with this reduced effective population size, our results suggest that the evolution of the Z chromosome in *S. japonicum* is dominated by the effect of relaxed purifying selection. This is in line with the general pattern of faster rates of evolution on Z chromosomes, which are often caused by drift, likely due to their smaller effective population size (Mank, Nam et al. 2010; Mank, Vicoso et al. 2010; Hayes et al. 2020; Chase et al. 2024; Mongue and Baird 2024). Although both the hemizygous and diploid Z regions are under reduced efficacy of purifying selection, we could to some extent test the differential expectations for the hemizygous and diploid X-linked loci by focusing on sex-biased genes. In the young diploid Z region, selective constraints should be relaxed for unbiased and female-biased genes due to the sheltering effect of functional gametologs on the W, and this effect should be stronger for genes expressed primarily in females (Mrnjavac et al. 2023). The effect of sheltering is supported by the fact that female-biased genes have the highest *π*_N_/*π*_S_, and the lowest inferred *α*, of the genes in the diploid Z region. However, increased *π*_N_/*π*_S_ is not reflected in increased *K*_a_/*K*_s_, possibly because this region stopped recombining only recently and there was not enough time for substitutions to accumulate under the new selective regime. Unbiased genes on young diploid Z region also show significantly higher *π*_N_/*π*_S_ than their autosomal counterparts, but the difference is less extreme than for female-biased genes, suggesting weaker effect of sheltering. As sheltering does not occur in males (males do not have W), we do not expect reduced efficiency of selection for male-biased genes on diploid Z. Similar *π*_N_/*π*_S_ levels for male-biased genes on diploid Z and autosomes support this prediction. Taken together, these results confirm that diploid and hemizygous sex-linked regions have different evolutionary dynamics, and that genes that function predominantly in one sex are primarily affected (assuming that sex-biased gene expression is a good proxy for sex-biased function).

Several theoretical models predict that Z chromosomes may become “masculinized” over time, i.e. they may lose genes with female-specific functions and gain genes that are expressed primarily in males (Gurbich and Bachtrog 2008; Mrnjavac et al. 2023). An excess of Z-linked genes of *S. japonicum* are indeed male-biased in their expression. In the hemizygous Z region, masculinized expression can to a large extent be explained by the incomplete dosage compensation system found in this group: the Z chromosome is upregulated in both sexes, and has higher expression in males, since males have two copies of the Z (Picard et al. 2018). Whether an ancestral enrichment in genes with male-specific functions favored the evolution of such an unusual regulatory mechanism has yet to be tested. Similar to Elkrewi et al. (2021), in the diploid Z region, expression patterns are largely similar to autosomal ones. This may reflect the young age of this nonrecombining region. If female-biased genes on the diploid Z remain under reduced efficacy of selection over sustained periods of time, they may eventually be lost or decrease in expression, potentially contributing to masculinization.

Our study illustrates different evolutionary dynamics of old and young sex-linked regions. Together with other studies on young sex-linked regions in butterflies of genus *Leptidea* (Höök et al. 2024), plant *S. latifolia* (Krasovec et al. 2018), and several *Drosophila* species (Nozawa et al. 2016; Nozawa et al. 2021), our study suggests that the reduced efficiency of selection due to sheltering might be widespread in young sex-linked regions. This body of work also illustrates the importance of studying nonmodel species, where diploid Z and X regions might be common, but underreported, as well as using population data for studying ongoing evolutionary processes.

## Methods

### Strata Determination

To identify hemizygous and diploid Z regions, we performed female-to-male coverage analysis and male-to-female *F*_st_ analysis as in Elkrewi et al. (2021), using the recently published male *S. japonicum* genome assembly (GCA_021461655.1) (Luo et al. 2022). Briefly, female (SRR6841388) and male (SRR6841389) *S. japonicum* reads were separately mapped to the *S. japonicum* male genome using *bowtie2* (Langmead and Salzberg 2012). Only uniquely mapped reads were kept. Coverage for male and female reads was calculated with *soap.coverage* (Luo et al. 2012) per 10,000 bp windows. Log2(F/M coverage) was calculated and visualized in R (R Core Team 2023). Coordinates of the hemizygous Z region were determined as the limits of the Z chromosome region where log2(F/M coverage) values are centered at −1, meaning there are twice as many reads in males compared with females (Z chromosome coordinates: 24470001-49640001).

The *F*_st_ analysis also followed the approach of Elkrewi et al. (2021), but using the new chromosome-level genome assembly. *F*_st_ between male and female reads (PRJNA650045, sex of the individual library was determined from Elkrewi et al. (2021)) was calculated with *vcftools* (Danecek et al. 2011) and visualized in R. The diploid Z region was determined as the region for which the male:female *F*_st_ values were consistently above the 95 percentile of the distribution across the genome (Z chromosome coordinates: 49640001-76240000). In this region 62.67% of windows had male:female *F*_st_ values above the 95 percentile of the genome-wide distribution and 90.42% of reads had male:female *F*_st_ values above the 90 percentile of the genome-wide distribution.

### Sex-biased Expression Analysis

Publicly available whole-body expression data was downloaded for 3 male and 3 female *S. japonicum* individuals at 28 d post-infection, (PRJNA343582, Wang et al. 2017). Gene expression levels were obtained per gene, per sample, using *kallisto* (Bray et al. 2016) and normalized with *sleuth* (Pimentel et al. 2017). Significant differential expression between males and females was tested with DESeq2 (Love et al. 2014). Genes with at least 2-fold difference in expression between males and females and *P*-value adjusted for multiple testing <0.05 were classified as sex-biased. Distributions of sex-bias in autosomes, hemizygous Z and diploid Z regions were visualized and compared in R. Sex-bias distribution was compared between the Z chromosome and autosomes with Mann–Whitney–Wilcoxon test.

### Divergence Inference

We identified orthologs between *S. japonicum* and closely related species *S. mansoni* (65% median synonymous divergence, Picard et al. 2018) as the best reciprocal blat (BLAST-Like Alignment Tool, Kent 2002) hits between *S. japonicum* and *S. mansoni* coding sequences (assembly version GCF_000237925.1, Protasio et al. 2012) (we chose the longest coding sequence per gene for the analysis). Orthologs were aligned using TranslatorX with the “gblocks” option (Abascal et al. 2010). Divergence between orthologs was calculated with *K*_a_Ks_Calculator 2.0 (Wang et al. 2010). Yang-Nielsen estimates of *K*_a_/*K*_s_ were obtained per gene, as well as the number of nonsynonymous and synonymous substitutions per gene. These parameters were visualized and compared in R. Boxplots represent the interquartile range (IQR) of the data with the median, while whiskers extend from the IQR to the largest value which is not further from the limits of IQR ± 1.5 IQR. Outlier values were excluded from the plots.

### Polymorphism Inference

We downloaded a publicly available population genomic dataset (PRJNA789681) from NCBI database (https://www.ncbi.nlm.nih.gov/), including whole genome sequences of 48 *S. japonicum* adult male individuals sampled from several locations in South-East Asia (we did not include the Taiwan and the Philippines subpopulations in our analysis as those subpopulations have extremely reduced levels of diversity and could have biased our analysis), corresponding to their worldwide range (Luo et al. 2022). We trimmed the reads with *Trimmomatic* (Bolger et al. 2014). Trimmed paired reads were mapped to the *S. japonicum* male genome assembly (GCA_021461655.1) using *bowtie2* with *–end-to-end* and *–sensitive* parameters, separately for every individual. Nonuniquely mapped reads were removed. SAM files were reformatted into sorted BAM files using samtools (Li et al. 2009). Variant calling was performed with *bcftools mpileup* option (Li et al. 2009), using (48) 72 individual bam files as input. Variants were filtered by quality, *bcftools view -i “%QUAL*≥*20”*, only biallelic sites were kept, *–max-alleles 2*, and indels were removed, *–exclude-types indels*. bcf file was reformatted into vcf file. Rare variants (maf < 15%) were removed with *vcftools* using *–maf 0.15 –max-missing 0.9* options. Polymorphic sites were annotated as synonymous or nonsynonymous using *snpEff* and *SnpSift* (Cingolani et al. 2012).

### Population Genomic Analyses

*α* denotes the proportion of nonsynonymous substitutions that are fixed by positive selection, and is based on the classic MK test. *α* per gene was calculated as 1−((number of nonsynonymous polymorphisms per gene (Pn)/number of synonymous polymorphisms per gene (Ps))/(number of nonsynonymous substitutions per gene (Dn)/number of synonymous substitutions per gene (Ds))) (Smith and Eyre-Walker 2002; Charlesworth and Charlesworth 2010, Chapter 6.4)), after removing variants below 15% frequency (Fay et al. 2001; Al-Saffar and Hahn 2022), using R. Distributions of *α* values for different categories of sex-bias, and different genomic regions: hemizygous Z, diploid Z and autosomal one, were visualized and statistically compared in R. Statistically significant differences between distributions were tested with the Wilcoxon–Mann–Whitney test.

In addition to *α*, we calculated DoS as DoS = Dn/(Dn + Ds)−Pn/(Pn + Ps) (Stoletzki and Eyre-Walker 2011). DoS is a measure of direction and degree of departure from neutrality, based on the MK test, that corrects for biases that arise from a small number of observations (Stoletzki and Eyre-Walker 2011). Statistically significant differences between distributions were tested with the Wilcoxon–Mann–Whitney test.

Nucleotide diversity along the genome, in 10,000 bp windows, was calculated using pixy (Korunes and Samuk 2021). Statistically significant differences between distributions in different genomic regions were tested with the Wilcoxon–Mann–Whitney test.

## Supplementary Material

evaf021_Supplementary_Data

## Data Availability

Publicly available data was used for this study. The data with the following accession numbers was downloaded from https://www.ncbi.nlm.nih.gov/. *Schistosoma japonicum* male genome assembly:GCA_021461655.1 (Luo et al. 2022). *Schistosoma japonicum* female whole genome sequences: SRR6841388. *Schistosoma japonicum* male whole genome sequences: SRR6841389. *Schistosoma japonicum* male and female whole genome sequences used for F_st_ analysis: PRJNA650045. *Schistosoma japonicum* male and female RNA sequences: PRJNA343582 (Wang et al. 2017). *Schistosoma japonicum* genome assembly: GCF_000237925.1 (Protasio et al. 2012). *Schistosoma japonicum* population genomic dataset: PRJNA789681 (Luo et al. 2022). Code used for this study and intermediate files can be found at: https://git.ista.ac.at/amrnjava/schistosomes_slower_z
