# Supplementary material for: Reduced Efficacy of Selection on a Young Z Chromosome Region of Schistosoma japonicum
Source: Genome Biol Evol. 2025 Feb 6;17(2):evaf021. doi: 10.1093/gbe/evaf021 (PMC11833683; doi:10.1093/gbe/evaf021)
Supplement: evaf021_Supplementary_Data [file evaf021_supplementary_data.pdf]

Supplementary figures

A)

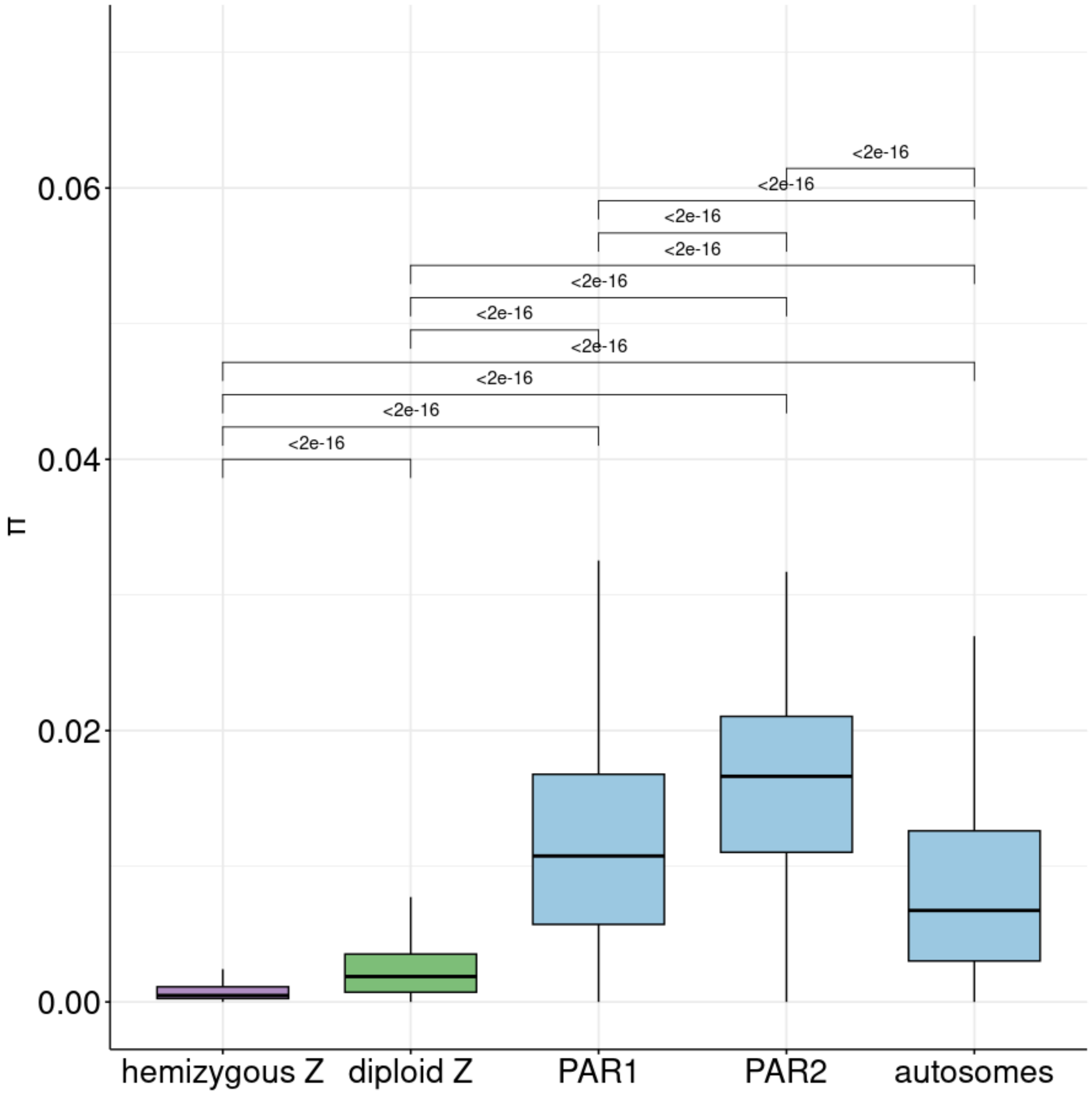

B)

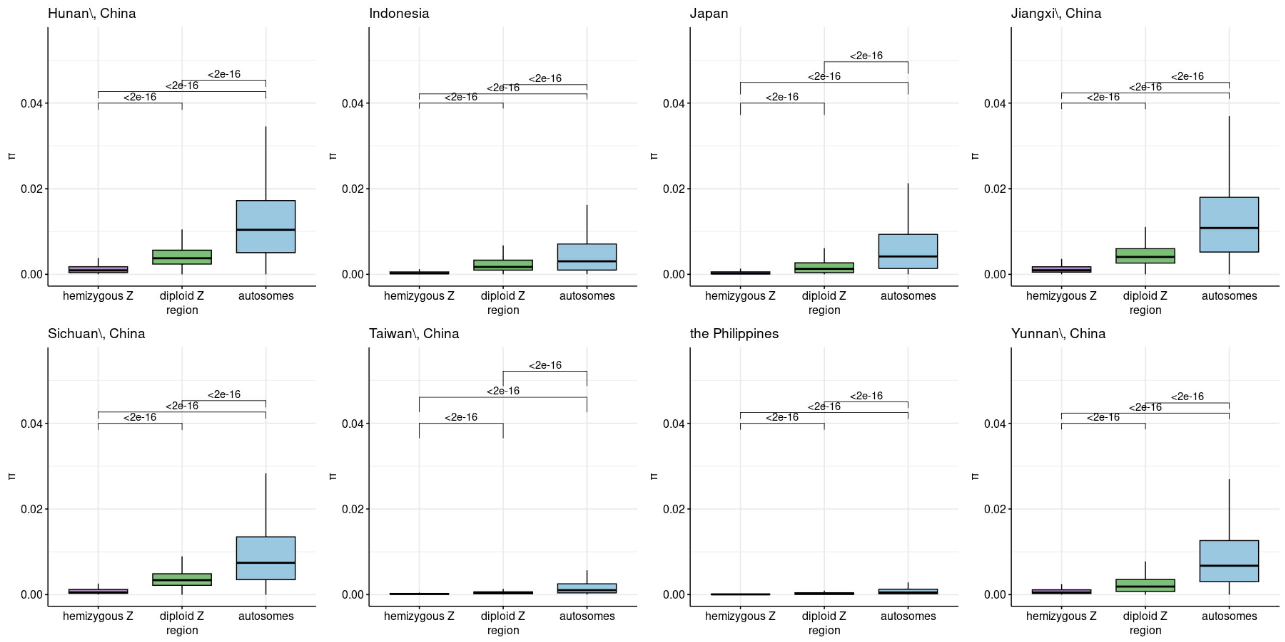

Supplementary Figure 1. A) Nucleotide diversity (π) of hemizygous Z, diploid Z and pseudoautosomal regions and autosomes. B) nucleotide diversity in subpopulations.

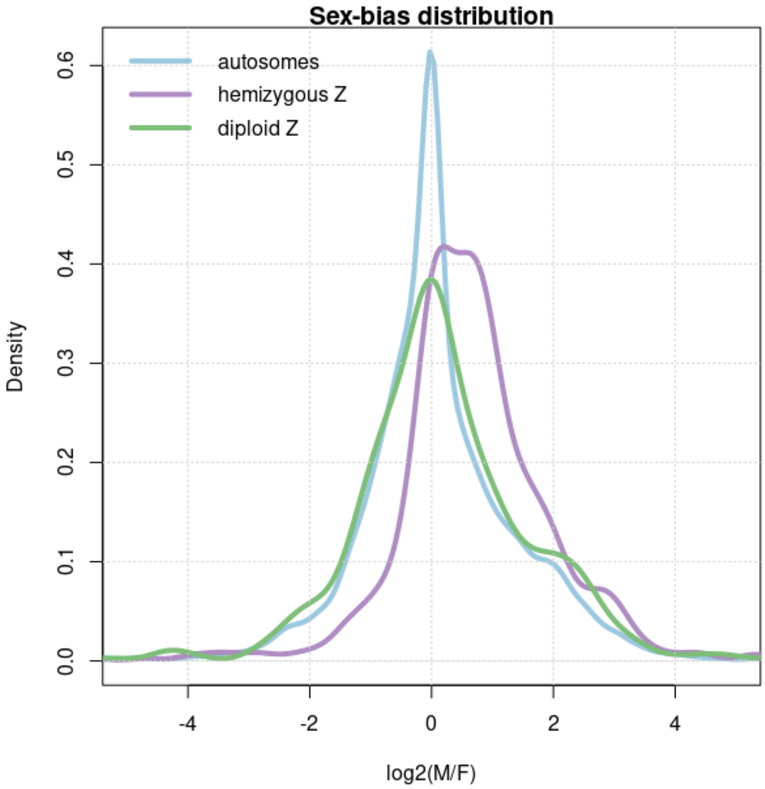

Supplementary Figure 2. Sex-bias distribution in autosomes, diploid Z region and hemizygous Z region.

A)

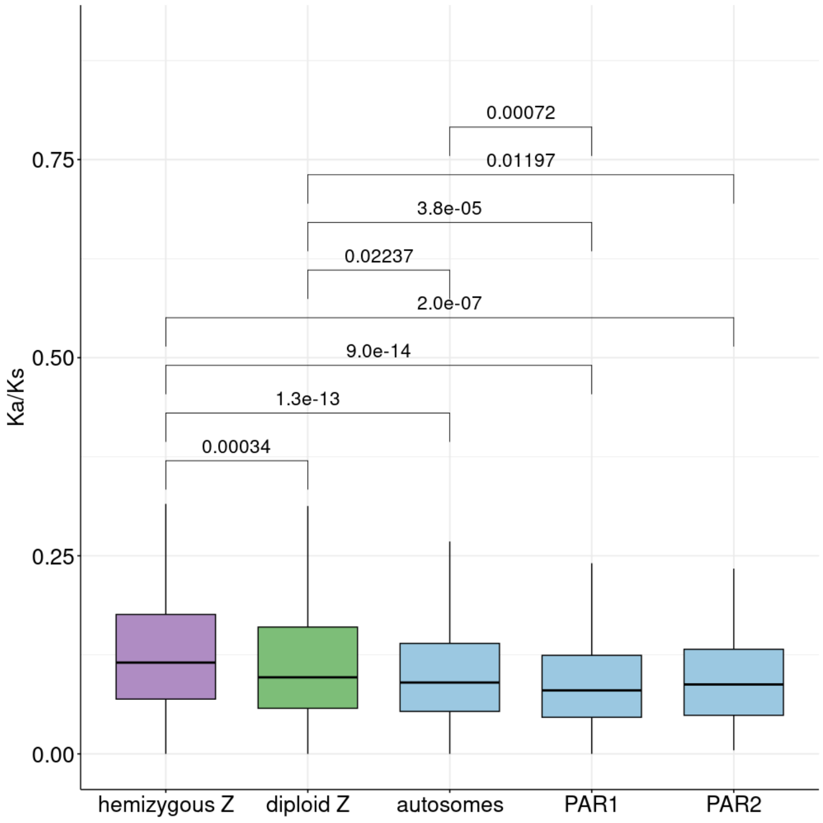

B)

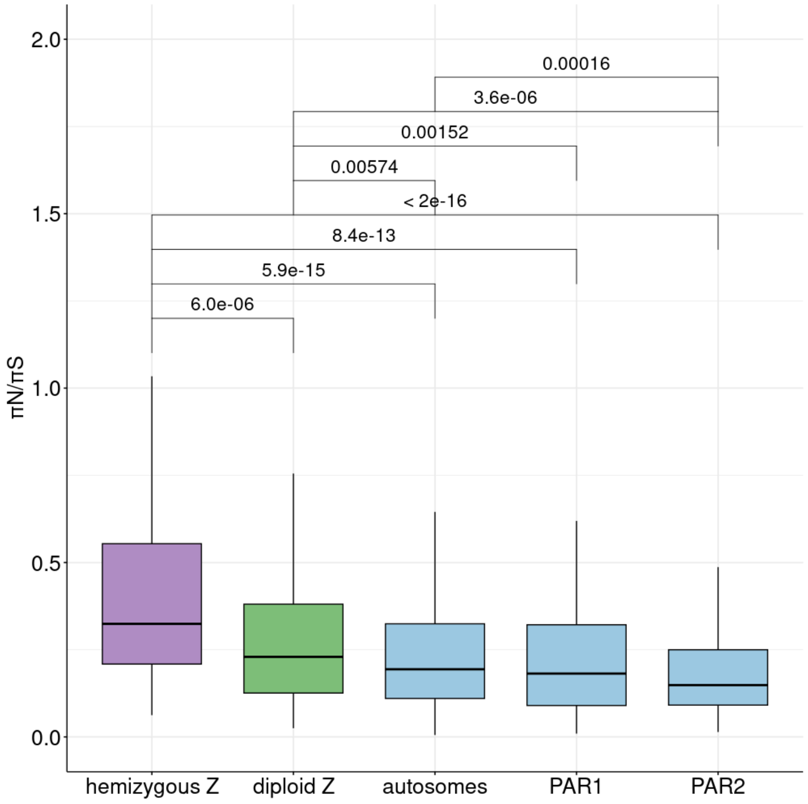

Supplementary Figure 3. K_a/K_s and π_N/π_S for hemizygous Z, diploid Z, autosomes and PARs

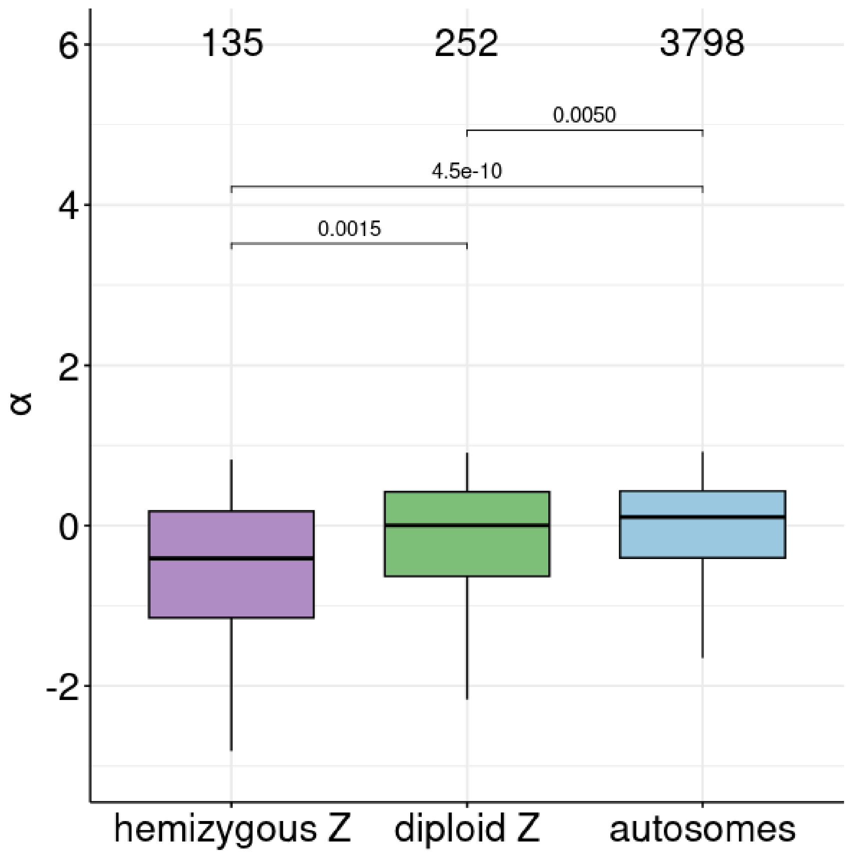

Supplementary Figure 4. α as a function of genomic location

A)

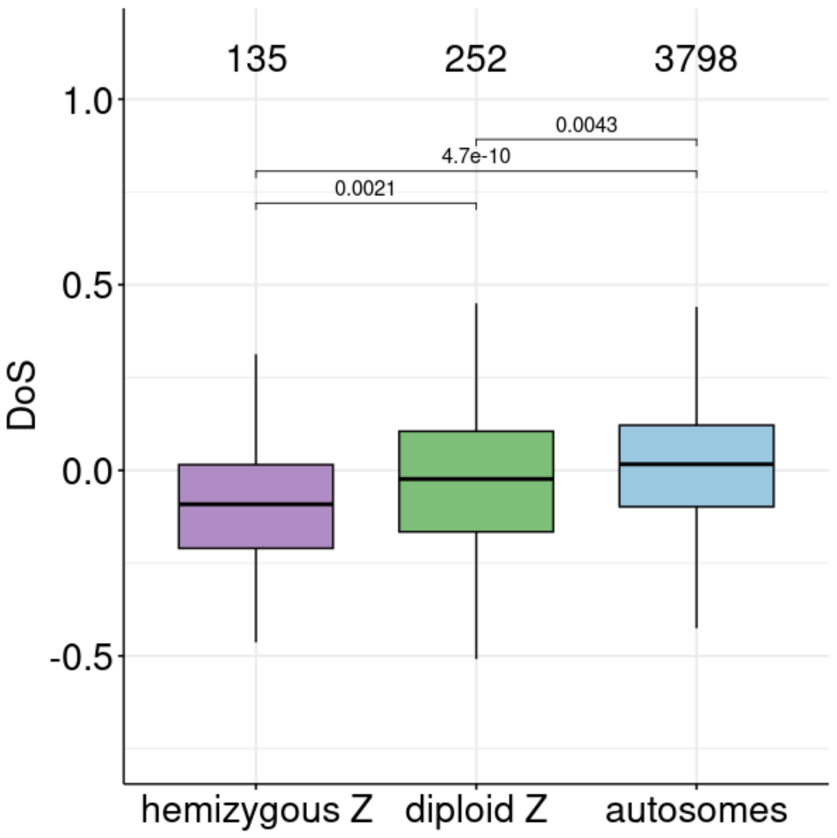

B)

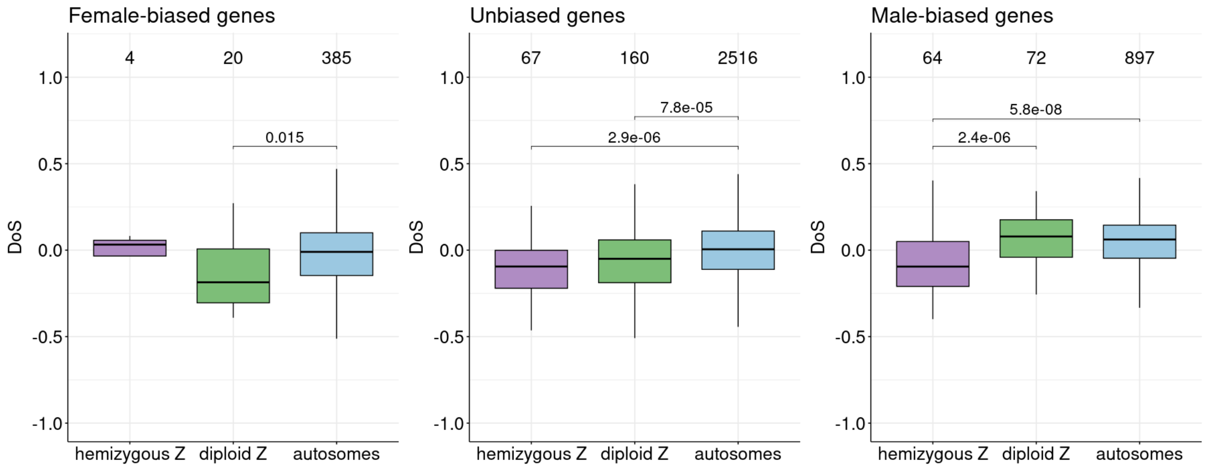

Supplementary Figure 5. **A**) Direction of Selection (DoS) as a function of genomic location **B**) Direction of Selection (DoS) as a function of sex-bias and genomic location. Numbers of genes are labeled above boxplots.

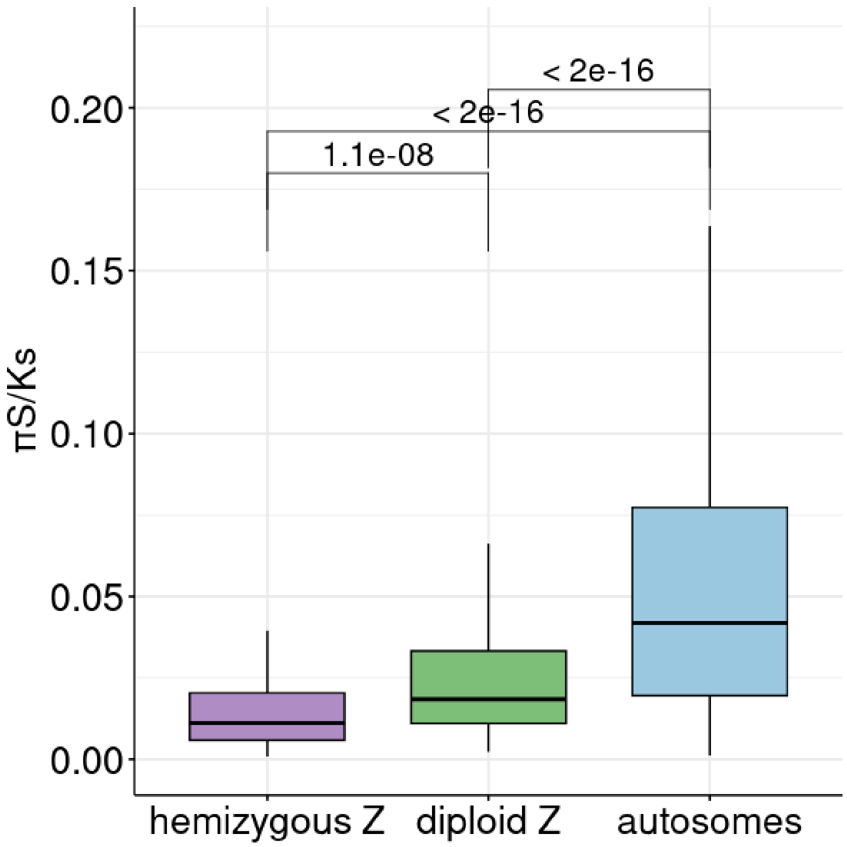

Supplementary Figure 6. πs/Ks for hemizygous Z, diploid Z and autosomes
